# Improving Thermo-Sealing of Poly(3-hydroxybutyrate-co-3-hydroxyvalerate) by Blending with Polycaprolactone

**DOI:** 10.3390/polym16233255

**Published:** 2024-11-23

**Authors:** Eva Moll, Amparo Chiralt

**Affiliations:** Instituto Universitario de Ingeniería de Alimentos (FoodUPV), Universitat Politècnica de València, Camí de Vera s/n, 46022 València, Spain

**Keywords:** PHBV, PCL, blended films, barrier properties, heat sealing

## Abstract

Poly(3-hydroxybutyrate-co-3-hydroxyvalerate) (PHBV) is a biodegradable biopolymer from the PHAs family that has potential to replace conventional plastics and reduce plastic pollution. However, PHBV has thermo-sealability issues, making it challenging to use for bags. Blending it with polycaprolactone (PCL) could address this but may alter the barrier properties of the films, affecting their effectiveness as food packaging material. This study examined the properties and heat-sealing capacity of PHBV/PCL blend films (ratios: 60/40, 50/50, and 40/60), obtained by melt blending and compression moulding. Both polymers are immiscible and were in separated phases; the continuous phase was PHBV in the 60/40 blend and PCL in the 40/60 blend, while the 50/50 sample exhibited interpenetrating bicontinuous phases of both polymers. The permeability to water vapour, oxygen, and D-limonene increased as the PCL content rose, especially when it formed the continuous phase in the matrix. The elastic modulus and resistance to break decreased, while extensibility increased, more markedly when PCL was the continuous phase. However, the continuity of PCL phase provided the films with better thermal adhesion and seal strength. The 50/50 blend showed the best balance between heat sealability and barrier properties, making it the most suitable for food packaging in sealed bags.

## 1. Introduction

Food packaging is essential for preserving food from physical, chemical, and microbiological deterioration, ensuring quality and safety for extended periods. This is especially important to facilitate global transport in current consumer habits and globalisation that involves the consumption of all types of food products, regardless of their origin [1]. Nevertheless, the widespread use of single-use non-degradable plastics, along with inadequate waste management, has created severe environmental problems, polluting terrestrial and, especially, aquatic habitats [2,3,4,5]. While some of these plastics can be recycled, many are difficult to process and often end up in landfills or are incinerated, contributing to air pollution and posing risks to public health [6,7,8,9].

Global production of fossil-based plastics continues to increase with 413.8 Mt in 2023 [10]. To mitigate the environmental problems arising from their lack of degradability and the use of non-renewable resources, the production of mechanically recycled plastics (from 30 Mt in 2018 to 36.2 Mt in 2023) and bioplastics (1.2 Mt in 2018 to 2.18 Mt in 2023) is also increasing, with very little production of chemically recycled and carbon-captured (0.1 Mt) plastics [11]. Global bioplastics production capacity is projected to increase significantly to approximately 7.43 Mt in 2028. Packaging is the largest market segment for bioplastics, holding 43% of the total bioplastics market in 2023 [10]. Dokl et al. [12] have analysed, on global and regional levels, the projections of the plastic consumption in different regions, considering the polymer types, their applications, and waste up to 2050, and conclude that proactive policies can mitigate sustainability challenges, but it is necessary to reduce the footprints associated with the energy and virgin plastic production and to promote the production of bio-based plastics and recycling.

The progressive replacement of traditional plastics by biodegradable materials in the food packaging field will contribute to addressing these problems [13,14]. Biodegradable polymers can come from different sources, both renewable and non-renewable. Among biopolymers from renewable sources, there are starch, chitosan, or some proteins such as casein, which come directly from biomass. Others, such as PLA (polylactic acid), can be synthesised from lactic acid produced in the fermentation of different carbon sources, such as starch. Another group, the polyhydroxyalkanoates (PHAs), are produced by numerous bacteria (more than 300 Gram-positive and Gram-negative types) and archaea under stress conditions [15]. Specifically, they are synthesised from carbon-rich residues but under limitations of basic nutrients like phosphorus, nitrogen, or oxygen, allowing microorganisms to synthetise the polymers and store them as repositories of carbon and energy [16,17,18,19,20]. Within the PHA family, polyhydroxybutyrate (PHB) stands out as a promising material for packaging applications. However, its highly crystalline structure makes it brittle and hard, limiting its practical use. Furthermore, its melting point is close to its degradation temperature, making thermal processing challenging. To address these limitations, PHB is often copolymerised with hydroxyvalerate (HV) to form poly(3-hydroxybutyrate-co-3-hydroxyvalerate) or PHBV [21,22,23]. This aliphatic copolymer is biosynthesised by bacteria from different substrates, such as waste from the food industry [24,25,26]. Depending on the carbon source used, the proportion of HV can vary, resulting in copolymers with different properties [22,27].

PHBV is insoluble in water, resistant to hydrolysis and UV degradation, and has a high degree of crystallinity. The latter translates into excellent oxygen and water vapour barrier properties (similar to PET) and balanced mechanical properties in terms of strength and stiffness [28]. However, the high crystallinity gives it an intrinsic brittleness that progresses over time due to physical ageing and recrystallisation. In addition, its processability is limited by the proximity of its degrading temperature and melting point, which hinders its industrial processing by reducing its processing window. Its brittleness, low impact resistance, thermal instability, and high production cost limit its suitability for packaging on its own [29]. Likewise, its low melt viscosity and low adhesion pose a challenge during heat-sealing and thermoforming processes, as the material softens too much at moderate temperatures [30]. Therefore, studies have been conducted to overcome its plastic deficiencies, such as blending with other more resistant or ductile polymers, such as polycaprolactone (PCL), to obtain new materials with improved properties compatible with industrial processes [31,32,33,34,35].

PCL is a biodegradable polymer from non-renewable resources (petrochemicals) that is hydrolytically degraded through the cleavage of its ester bond by the enzymatic action of fungi and bacteria, although its degradation is slow due to its long aliphatic chain and high degree of crystallinity [36]. This semi-crystalline aliphatic polymer exhibits a low glass transition temperature (about −60 °C) and a very wide window for thermoprocessing, as its melting point is around 60 °C and degrades at more than 350 °C [37,38]. This represents a decisive factor in the sealability and could favour the sealing of its blends with other polymers, such as PHBV. PCL shows good miscibility with a wide variety of polymers, but its lack of compatibility with PHB has been reported [39]. Therefore, mixing conditions are a key factor to ensure a good interaction between the two polymers in the blend [31].

In order to improve the toughness of PHBV, PHBV/PCL blends have been previously studied, analysing their miscibility, thermal properties, in vitro degradation, and mechanical properties [32,40]. These studies have shown that PHBV and PCL are immiscible, and to promote blending, different strategies have been applied to modify the crystalline topology of the blends, such as the use of plasticisers or compatibilisers, promoting the interfacial adhesion of both polymers, their uniform dispersion, and their improved toughness, hardness, and strain at break. In general, PHBV/PCL blends have been found to have several advantages, such as biodegradability and biocompatibility with biological tissues. The presence of PHBV in the blends improves the barrier properties of the material, which is essential in developing food packaging systems. In addition, they are more flexible, with better processability by various techniques (extrusion, electrospinning, 3D printing…) [32,41]. No previous studies were found on the heat-sealing capacity of PHBV–PCL blend films, with this aspect being crucial for food packaging applications.

For the purpose of obtaining a homogeneous film seal and a good sealing of the container, a sufficient proportion of PCL, which is mainly responsible for heat sealing, must be present throughout the package closure. Likewise, PCL distribution in the blend matrix is also an important factor to ensure homogeneous sealing. Nishida et al. [42] reported that in PHB-rich blends, PHB was the continuous phase with dispersed PCL domains, whereas in PCL-rich blends, the continuous phase was PCL with dispersed PHBV regions. At a 1:1 PHB/PCL ratio, the matrix showed a bicontinuous morphology with interpenetrating continuous phases of both polymers. Therefore, blends having a near 1:1 PHBV/PCL ratio are expected to have the best heat-sealing ability.

The aim of this study was to obtain PCL and PHBV blend films with different ratios (near 1:1) by melt blending and compression moulding in order to analyse their heat sealability, and thus, their ability to produce bags for food packaging applications. The films’ mechanical, thermal, and optical properties, as well as their microstructure, were examined, with a special emphasis being placed on their barrier properties, which are particularly important for food packaging. These evaluations will support their potential as food packaging materials, ensuring effective sealing, suitable barrier capacity, and adequate mechanical performance.

## 2. Materials and Methods

### 2.1. Materials

Poly(hydroxybutyrate-co-hydroxyvalerate) or PHBV was purchased from TianAn Biologic Materials (Ningbo, China) with an HV ratio of 2%, while polycaprolactone with Mn 80,000 Da was procured from Sigma-Aldrich Chemie (Steinheim, Germany). To keep the films moisture-controlled, P_2_O_5_ and Mg(NO_3_)_2_ were used as appropriate.

### 2.2. Obtaining Films

The films were obtained according to the procedure previously described [21], with some variations. In brief, the PHBV dried in the oven for 24 h at 60 °C was blended with PCL in an internal mixer (Haake PolyLab QC, Thermo Fisher Scientific, Dreieich, Germany) for 7 min at 170 °C and 50 rpm in different ratios. Thus, 40, 50, and 60 g of PCL per 100 g of the mixture were used. The samples were named as 60/40, 50/50, and 40/60, which indicate the PHBV/PCL ratio. The blend mass was cold milled using liquid nitrogen in a Thermomix TM-5 device (Vorwerk Spain M.S.L., S.C., Madrid, Spain) and conditioned at 0% relative humidity (RH) in desiccators containing P_2_O_5_. The films (3.5 g of the powder per film) were thermoformed in a hot plate press (Model LP20, Labtech Engineering, Bangkok, Thailand) in three steps: (1) preheating at 180 °C for 5 min, (2) thermocompression at 180 °C for 4 min and 100 bars, and (3) cooling to about 60 °C in 4 min. Pure PHBV and PCL films were also obtained for comparison purposes using the same conditions for PHBV and 120 °C for PCL compression moulding. The films were stored at 0% RH until use.

### 2.3. Characterisation of the Film Properties

#### 2.3.1. Thermal Analysis

The phase transitions occurring in the samples as a function of temperature, as well as their thermal stability, were characterised by differential scanning calorimetry (DSC, STARe System, Mettler-Toledo Inc., Greifensee, Switzerland) and a thermogravimetric analysis (TGA 1, STARe System Analyser, Mettler-Toledo Inc., Greifensee, Switzerland), respectively. In both analyses, about 10 mg of samples previously cut into small pieces and preconditioned at 0% RH in P_2_O_5_ were used. For DSC analyses, the samples, placed in sealed aluminium pans, were heated from −40 °C to 200 °C at 10 K/min, maintaining this temperature for 2 min, then cooled to −40 °C at 10 K/min and held for 2 min, and then heated again to 200 °C at 10 K/min. For TGA analyses, the samples, in alumina crucibles, were heated from room temperature to 600 °C at 10 K/min under a N_2_ flow of 10 mL/min. Each analysis was performed in duplicate in each sample.

#### 2.3.2. Microstructure

The cross-section of the films and heat-sealed samples was observed by field emission scanning electron microscopy (FESEM, Ultra 55, Zeiss, Oxford Instruments, Abingdon, UK). For this purpose, the samples, conditioned at 0% RH, were cryofractured with slush N_2_, coated with platinum, and observed in the microscope at 2.00 kV.

#### 2.3.3. Barrier and Tensile Properties

The permeability to water vapour, oxygen, and D-limonene (as a model for aroma compounds) of the obtained films was measured. Water vapour permeability (WVP) and D-limonene permeability (LP) was measured according to the standard ASTM gravimetric method, ASTM E96-95 [43]. For WVP, 5 mL of distilled water was placed in a Payne permeability cup and capped with the films (3.5 cm in diameter), conditioned at 53% RH for 72 h, with previous measurement of their thickness at six random points with a digital micrometre (Comecta S.A., Barcelona, Spain). The assembly was placed in desiccators containing oversaturated Mg(NO_3_)_2_ solution at 25 °C. The cups were weighed periodically every 2 h for 48 h when the steady state was reached. The same procedure was used to measure LP, but introducing D-limonene into the Payne cups instead of water. This test was carried out for about 90 h. In both cases, the tests were carried out in triplicate for each formulation, and the respective water or D-limonene transmission rate was determined from the slope of weight loss vs. time data. The permeability values were determined from the transmission rate considering the vapour pressure gradient through the film and its thickness.

To determine the oxygen permeability of the films, the ASTM D3985-05 method [44] was followed, using an oxygen permeation analyser (Model 8101e. Systech Illinois, Thame, UK). The samples conditioned at 53% RH, with measured thickness, were cut according to the standard of the equipment (area: 50 cm^2^) and measured. The oxygen transmission rate (OTR) was measured every 15 min until equilibrium was reached. The OP of the films was calculated considering the oxygen pressure on both sides of the film and its thickness. The measurements were carried out in duplicate for each sample.

The film tensile properties were obtained according to the standard method ASTM-D882 [45]. Sample strips (25 × 100 mm^2^) were cut (12 per formulation) and conditioned at 25 °C and 53% RH after measuring their thickness at 6 random points. The samples were clamped (5 cm separation) in the grippers of the universal test machine (TA-XTplus model, Stable Micro Systems, Haslemere, UK) and stretched at 50 mm/min to obtain the tensile stress–strain curves. From each curve, the elastic modulus (EM), tensile stress at break (TS), and elongation at break (%E) were obtained.

#### 2.3.4. Optical Properties

A CM-5 spectrocolourimeter (Konica Minolta Co., Tokyo, Japan) was used to analyse the colour and internal transmittance of the films. For this purpose, the reflection spectra of the films (from 400 to 700 nm) were obtained on a black and white background, and the multiple scattering theory of Kubelka–Munck was applied to obtain the internal transmittance (Ti) and reflectance of an infinite thickness film (R_∞_) [46]. The latter was used to determine the CIEL*a*b* colour coordinates, considering the D65 illuminant and 10° observer [47]. From the a* and b* coordinates, the chroma (C_ab_*) and hue (h_ab_*) of each film were calculated. Each film was measured in triplicate in three films per formulation.

#### 2.3.5. Fourier Transform Infrared (FTIR) Spectroscopy

Fourier transform infrared spectroscopy in the attenuated total reflectance mode (FTIR-ATR) was used to characterise the films. The FTIR-ATR spectra (4000 and 500 cm^−1^) were obtained in the wavelength range of 4000–650 cm^−1^ at a resolution of 6 cm^−1^, applying 128 scans for each spectrum, using a molecular spectrophotometer (FTIR 630 Diamante, Agilent Technologies, Santa Clara, CA, USA) equipped with an attenuated total reflectance accessory. Four measurements were performed on three different films for each formulation.

#### 2.3.6. Determination of the Sealing Strength

To determine the sealing strength of the films, two strips of each formulation were adhered together, and the seal strength was measured according to the methodology described in the ASTM F88/F88M-23 [48] method using the universal test machine (TA-XTplus model, Stable Micro Systems, Haslemere, England). For this purpose, films strips (10 × 2.5 cm) were prepared and conditioned at 53% RH for one week. Pairs of strips of each formulation were sealed at the end in an area of 2.4 × 2.5 cm. The strip pairs of each formulation were sealed at the edge in an area of 2.4 × 2.5 cm, according to the standard, using the hydraulic hot plate press (LP20, Labtech engineering, Bangkok, Thailand) at 120 °C for 30 s. These conditions were selected based on previous trials, considering a temperature above the melting point of PCL and below the PHBV melting. Thus, the molten PCL flow through the sealing region would be responsible for the heat sealing, avoiding the problems of excessive fluidity of the molten PHBV. The free parts of the sealed strip were clamped in the grippers with 50 mm separation and stretched at a grip separation speed of 200 mm/min. The seal strength was obtained, as described in the standard, through the mean force value in 80% of the points (central data) of the force–distance curves by applying Equation (1):

(1) $$Seal\;Strenght=\frac{Mean\;Force\;(N)}{Film\;width\;(m)}$$

#### 2.3.7. Statistical Analysis

To analyse the significant differences between the samples, an analysis of variance (ANOVA) test was performed with a Fisher’s least significant difference of 95% confidence. These differences are marked with different letters in the tables. Statgraphics Centurion XVIII software (Statgraphics Technologies, Inc., The Plains, VA, USA) was used for this purpose.

## 3. Results and Discussion

### 3.1. Morphology and Structure of Films

**Figure 1:**
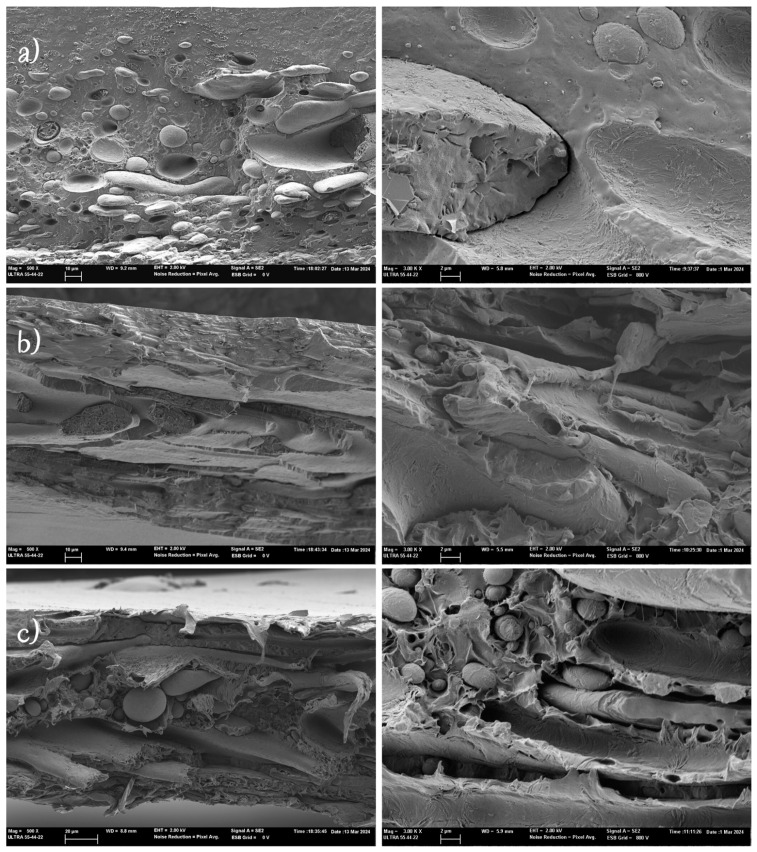
FESEM images of the cross-section of the PHBV/PCL blend films: (**a**) 40/60, (**b**) 50/50, and (**c**) 60/40 (**left**: 500×, **right**: 3000×).

The cross-section images of the films obtained from FESEM are shown in Figure 1 at different magnification levels. The images reveal the lack of miscibility between the two polymers, previously described by other authors for PHB-PLC blends [31,39,49]. Polymers were in separate phases in the films, with different dispersion characteristics depending on their ratio. Emergent particles or the voids left by the initially present particles are mainly observed in the micrographs, which suggests a low interfacial bonding between both dispersed and continuous phases. Likewise, the gabs between particles and the surrounding phase also point to scarce adhesion forces at the interface. In the 60/40 blend, a high dispersion of spherical or elongated PCL particles of different sizes can be observed embedded in a continuous PHBV matrix. The elongated shapes are the result of the blending process, which causes the dispersed material to be stretched by the shear forces of the rotor, resulting in the partial fragmentation of the PCL into smaller spherical particles. By increasing the PCL ratio up to 50/50, PCL and PHBV interpenetrating layers can be observed, showing a hindered polymer dispersion and continuous phase definition. This kind of structural arrangement was also observed by Nishida et al. [42] in 1:1 PHB-PCL blends and was described as a bicontinuous morphology, with both polymers exhibiting interpenetrating continuous phases. However, in the samples with a 40/60 ratio, containing the highest proportion of PCL, the typical stringy fracture of PCL [50] can be observed in continuous sheets, entrapping particles of the PHBV, which now constitutes the dispersed phase. Other authors [31] also observed the formation of a continuous PCL phase for PCL ratios above 50% in the PHBV/PCL blend films. This structural change will have a notable impact on the physical properties of the blend films and their ability for heat sealing.

**Figure 2:**
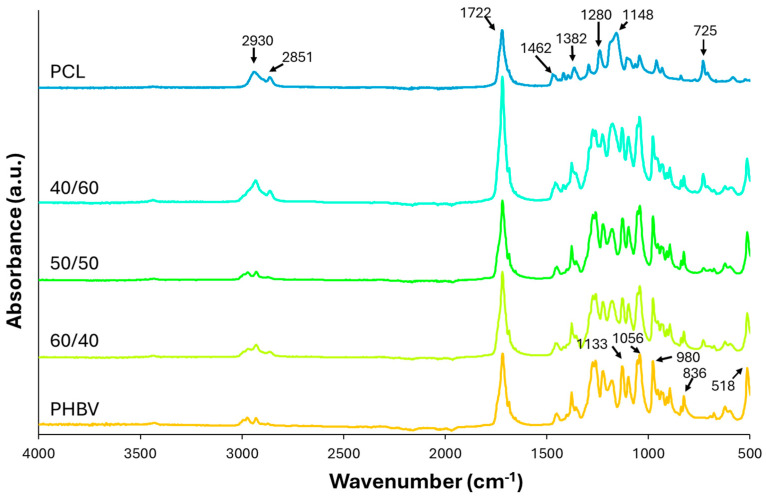
FTIR spectra of pure PCL and PHBV and of the different blends obtained.

The FTIR transmission spectra in the 500–4000 cm^−1^ region of the blends are shown in Figure 2, together with those of the pure polymers. All of the spectra show the peak at 1722 cm^−1^, associated with the vibration of the carbonyl group [51,52,53] present in both polymers. The peaks at 2930 and 2851 cm^−1^, representing the asymmetric and symmetric CH_2_ stretching, respectively, refs. [51,54] are more pronounced in PCL and also appear in pure PHBV and blend films. The C-H stretching vibration, reflected in the peaks between 836 and 980 cm^−1^, was more pronounced for PHBV, and the peaks at 1382 and 1462 cm^−1^, attributed to C-H bending vibrations, were more intense in PCL [47,55]. Peaks at 1280, 1133, and 1052 cm^−1^ are also observed in both polymers, associated with the C-O and C-C stretching in the crystalline phase and the C-O-C asymmetric stretching bands, respectively. The peak at 1148 cm^−1^, characteristic of the C-O-C symmetric stretching band, also appears in both polymers [56]. Nevertheless, differences in the FTIR pattern of both compounds can be observed and reflected in the spectra of the blend films, which show the overlapping bands of the individual polymers in a similar proportion to that of the polymers in the blend without a significant shift of the typical peaks of each polymer. This agrees with the lack of miscibility of both polymers with no specific interactions between chains that could change the FTIR spectra pattern in the blends.

### 3.2. Thermal Behaviour

**Figure 3:**
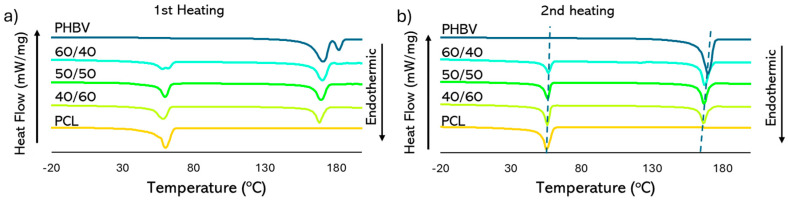
DSC thermograms of the (**a**) first and (**b**) second heating of the pure polymers (PCL and PHBV) and PHBV/PCL blends at different ratios: 60/40, 50/50, 40/60.

The polymer crystallisation behaviour in the PHBV/PCL blends was studied by a DSC analysis and were compared with those of the pure polymers. Figure 3 shows the thermograms corresponding to the first and second heating steps, with the thermal history of the polymers being deleted in the second one. Table 1 shows the melting peak temperature and enthalpy obtained from the thermograms and the degree of crystallinity calculated of each polymer, considering the polymer mass fraction in the film and the ΔH_m_ values of completely crystallised PHBV (ΔH_0_PHB = 132 J/g polymer, [57]) or PCL (ΔH_0_PCL = 139.3 J/g polymer, [58]). All blends clearly show two melting peaks, associated with the separated melting of each polymer in the blend, also demonstrating their immiscibility [32,59]. The peak around 60 °C corresponds to the PCL melting while the second, at about 170 °C, is attributed to the PHBV melting. Previous studies [60] also observed this crystallisation behaviour for PHBV–PCL blend films, associated with the fact that each polymer crystallises in separated phases with their typical crystalline structure, as also deduced from the FESEM images. However, some differences can be observed in the blends due to the crystallisation interferences between the polymers. Pure PHBV showed double melting peaks on the first heating, which can be attributed to the polymer recrystallisation during the annealing upon heating [61,62]. The smaller crystals melt at lower temperature and recrystalise in bigger crystalline forms that melt at higher temperature. This phenomenon did not occur in the second heating scan of pure PHBV and must be attributed to the faster cooling of the films after the thermocompression step (about 30 °C/min) compared with the DSC cooling step (10 °C/min), which promotes the formation of small spherulites. This recrystallisation process during the first heating step was not observed in the PHBV–PCL blend films, which suggests that the presence of PCL inhibited this phenomenon, while slightly reducing the melting temperature of PHBV, especially when its ratio rose in the blend. This reduction was also observed in the second heating step of DSC and suggests that the interferences of both polymers in the blends provoked a diminution in the crystal sizes of PHBV while increasing those of PCL, as its T_m_ values slightly rose as the PHBV ratio increased.

The melting enthalpy (ΔH_m_) and degree of crystallinity (χ_c_) of polymers in the blends were also slightly affected by the blending effect. For PHBV, melting enthalpy was lower in the blends than in pure polymer, whereas this only occurred for PLC at the highest ratios of PHBV. Likewise, the peak crystallisation temperatures (Tc), determined in the cooling step, of pure polymers were 27.4 and 122.3 °C, respectively, for PCL and PHBV, exhibiting the supercooling effect to be associated with the initial nucleation. The differences between melting and crystallisation temperatures (ΔT) were 28 and 47 °C, respectively, for pure PCL and PHBV. In all blends, supercooling decreased for PCL (ΔT = 20 °C), whereas it increased for PHBV (ΔT = 56 °C). This suggests that PHBV crystallisation was inhibited by the presence of liquid PCL in the blend, whereas the already formed crystals of PHBV promoted the PCL crystallisation rate, acting as nucleating agents. All of this is coherent with the differences in T_m_ and ΔH_m_ with respect to the pure polymers commented on above. As suggested by other authors [63], the lower crystallisation rate of PHBV in the blends could be due to the hindering effect provoked by blending. This is further reflected in the lower melting temperature in the blends, which decreased as the proportion of PCL rose, indicating the formation of smaller crystals that melt faster. Some authors reported that increasing the proportion of PCL in the blends decreased the crystallisation rate of PHBV, as the primary nucleation of the polymer is strongly affected by the presence of another compound [40]. The presence of PCL could be an impediment to the PHBV spherulitic growth as crystallisation is strongly influenced by the level of phase separation prior to its formation; the greater the separation of the components, the lesser the mutual impact on their crystallisation. In contrast, PCL crystallisation in the blends was favoured by the nucleating effect of the PHBV crystals formed at higher temperature during cooling.

As reported in other studies [32,63], the glass transition temperature of the amorphous phase (Tg) could not be detected due to the weak PHBV signal and the low Tg of PCL (below −20 °C; [64]). Nevertheless, thermo-mechanical analyses [31] revealed that the glass transition temperature of the polymers in the blends were slightly displaced with respect to the pure polymers, suggesting some polymer miscibility within the amorphous phase. Cavalcante et al. [65] concluded that interactions between PCL and PHB occurred preferentially in the chain segments located between the crystals of the polymers by applying time-domain nuclear magnetic resonance (TD-NMR). These authors observed the miscibility of PHB and PCL chains in areas with thicknesses around 30 nm in 90:10 PHB/PCL blends.

**Figure 4:**
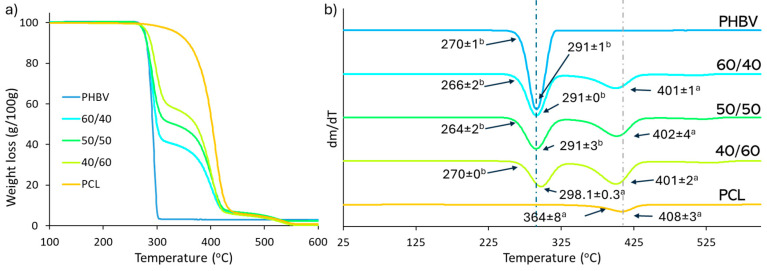
(**a**) TGA and (**b**) DTGA curves of PHBV/PCL blends with different ratios and the pure polymers with the initial degradation temperature and maximum rate temperature for the different degradation steps. Different superscript letters (a,b) indicate significant differences among formulations (*p* < 0.05).

The thermal stability of the polymers in the blends was analysed trough the TGA curves and its derivatives DTGA, shown in Figure 4a and Figure 4b, respectively. It can be clearly seen that PCL and PHBV have two separated degradation steps, coherently with their immiscibility, at the typical temperatures of each polymer [31,49], with the PHBV being less thermostable than PCL. The two peaks in the DTGA curves show the temperature of maximum degradation rate (T_p_) of each polymer, which were very close to those corresponding to the pure polymers. However, a small shift of the T_p_ of PHBV to higher temperatures was observed as the PCL ratio rose while the T_p_ of PCL slightly deceased in the blends. This behaviour also points to slight interactions between the polymers in the blend, as also described in previous studies [31,39].

### 3.3. Mechanical, Barrier, and Optical Properties

PHBV exhibits good gas barrier properties, better than other biopolyesters such as PCL and petroleum-based polymers [66], mainly due to its high crystallinity, as mass transport is highly hindered in the crystalline domains [28]. Therefore, blends with other polymers may modify these properties, decreasing its barrier capacity, with a detrimental effect for packaged food preservation. Table 2 shows the permeability to water vapour, oxygen, and aroma compounds (D-limonene) of the blend materials in comparison with pure polymers. Pure PCL films exhibited much higher permeability to water vapour, D-limonene, and oxygen than PHBV films, as previously reported [67]. The WVP and OP values obtained for pure PCL and pure PHBV were in the range found by other authors [67,68] considering the variability associated with the polymer characteristics and the film processing method. The aroma permeability (LP) was in the range determined by using the same method for PHBV and PCL films obtained by casting [69] and for thermoprocessed PHB-PCL (80: 20) blend films (90 g/m·s·Pa) [67] and increased when the PCL rose in the mixed films due to the higher LP values of PCL. Therefore, PCL worsened the barrier performance of blend films with respect to pure PHBV films. As shown in Table 2, a progressive increase in the permeability values was observed as the PCL rose in the blend. This was more marked for WVP and LP, especially for blends 50/50 and 40/60, where PCL acquires continuity in the film matrix. Surprisingly, the OP values varied in a narrower range despite the high OP values of PCL. When PCL was dispersed in the PHBV network (sample 60/40), a low effect of PCL on the film barrier capacity was produced due to the fact that it acts as a filler in the PHBV matrix with lower contribution to the mass transfer rate. In this case, the dispersed, more permeable particles do not introduce an effective tortuosity factor for mass transport but only a small accelerating effect. In contrast, when PCL was continuous in the film matrix, the mass transport was faster due to its high permeability, but the PHBV domains introduce an effective tortuosity factor for mass transport due to its higher resistance (lower permeability) to mass transport. The lower effect of the blend on the OP values could be attributed to a high ratio of the PHBV filler and the more dispersed PHBV crystalline regions (smaller spherulites) in the composite films that could promote the effective tortuosity factor for the transport of oxygen molecules.

The tensile properties of PHBV were also affected by the presence of PCL in the blends, as shown in Table 2. Both the elastic modulus and resistance to break decreased when the PCL ratio rose, while the blend films became more extensible. This effect was more marked when PCL was the continuous matrix and PHBV was the dispersed phase (samples 50/50 and 40/60), as a greater influence of continuous PCL in the mechanical response of the films is expected. Other studies reported similar behaviour for PHBV/PCL blend films obtained by solvent casting [41,60] and melt blending [49]. PCL gave rise to soft and flexible films, unlike PHBV, so it can act as an impact modifier reducing the stiffness of blend films and providing them with higher flexibility and ductility [31]. In fact, PCL films did not break during the tensile test. Nevertheless, their extensibility was greatly reduced when a high ratio of PHBV was dispersed in the matrix due to its rigidity and brittleness that limit the elongation capacity of the continuous matrix.

**Figure 5:**
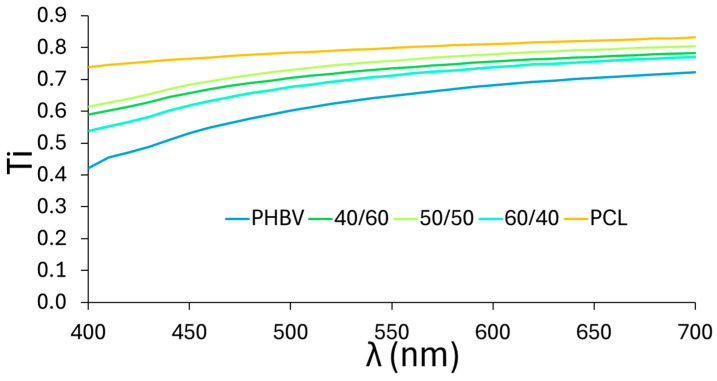
Internal transmittance spectra of pure PHBV and PCL films and of blends in different proportions of PHBV/PCL.

Concerning optical properties, PCL films were more transparent than PHBV films, as shown in Figure 5, where the internal transmittance spectra are plotted, showing the highest Ti values for the PLC films. As expected, films obtained from blending these two polymers have an intermediate internal transmittance. Although a higher proportion of PCL should increase Ti, the structural effects also play an important role. The high ratio of dispersed phase present in the 60/40 (PCL dispersed) and 40/60 (PHBV dispersed) blends promoted the light scattering and opacity of the material. In contrast, the 50/50 blends, with a bicontinuous phase of both polymers, were the least opaque, followed by the sample 40/60 with a continuous PCL matrix.

The same trend was observed in the colour coordinates (lightness, hue, and chrome) shown in Table 3. The most transparent PCL films showed the highest lightness while the pure PHBV films were the darkest, and the lightness of blend films ranged between those of the pure films. No significant differences were observed between PHBV films and the 60/40 blend, nor between the 50/50 and 40/60 blends. Nevertheless, there were significant differences between these two groups as the formation of a PCL continuous phase promoted the film lightness. The chromatic parameters of the blend films (C_ab_* and h_ab_*) were closer to those of PHBV, being yellowish and more saturated in colour in contrast with PCL films, which exhibited a very low saturated bluish hue. The total colour difference (ΔE) between the blends and PHBV was within the range of visual perception (2.0–4.7). These optical parameters, especially transmittance, could affect the degradation of photosensitive substances in packaged foods [70].

### 3.4. Heat Sealing of the Films

To ensure the quality and safety of food products from the point of origin to final consumption, protecting them from environmental factors such as external gases or micro-organisms, it is essential that the packaging containing the food is perfectly sealed [71]. During sealing, the polymer chains that make up the container must diffuse across the interface and interlock, closing it hermetically. It was not possible to seal the pure PHBV films at temperatures below and above its melting point due to its high crystallinity and excessive fluidity of its molten form, as observed in previous studies [72,73]. The applied sealing conditions to the PHBV/PCL blends were similar to those applied by Barisova et al. [74] to produce electrospun mats of PHB/PCL from dual-jet electrospinning of their separate spinning solutions, where the “segment” sealing was produced after heating the mats above the melting temperature of PCL, sealing the PHB fibres by the molten PCL fibres. As in this case, the PCL melts during the heating of the film strips, promoting the intertwining of chains in the heat-sealing area, while the PHBV remained solid. Thus, PCL, with a low melting temperature, facilitates the sealing of the blended films, while the solid PHBV helps to maintain the viscosity of the partially melted material.

**Figure 6:**
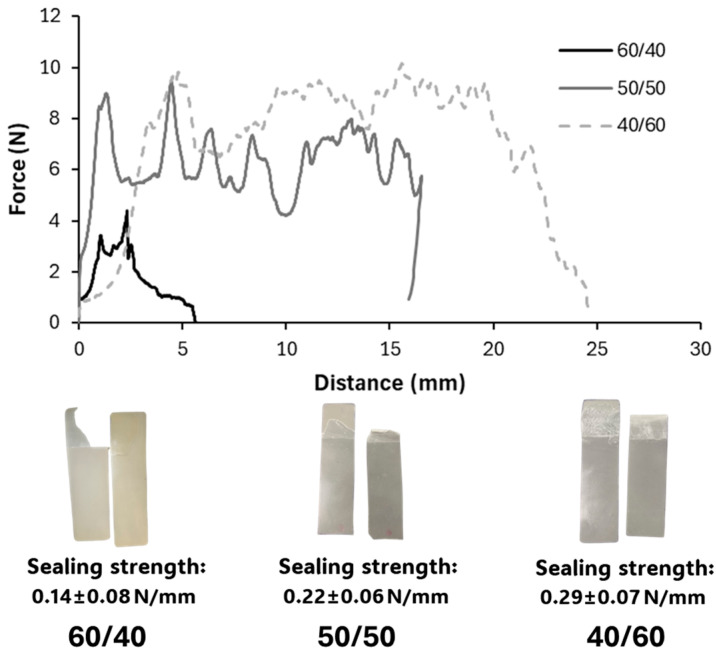
Typical force profile of the sealed film separation in the seal strength tests of blends 60/40, 50/50, and 40/60, showing the mean values ± standard deviation of seal strength (N/mm) and the typical peeling behaviour of the sealed films.

Figure 6 shows the typical force–distance curves obtained for the sealed strip separation of each blend formulation. The heat-sealed films showed good adhesion, with a seal strength increase as the PCL content rose in the film. The values ranged between 0.14 and 0.29 N/mm, which were below that of typical polyolefin-based seal layers but similar to some biodegradable polyesters, such as PLA, and lower than PBS, which exhibits a very good heat sealability [72]. The film samples where PCL was a continuous phase (50/50 and 40/60) showed greater seal strength than the 60/40 sample, which is coherent with the better contact between the PCL phases of both layers, favouring the chain interlocking. All samples showed a tear seal failure as the partially peeled foil broke during the seal strength test. This can be attributed to the high crystallinity of the material and its low extensibility, which could be enhanced by annealing during sealing. Najarzadeh et al. [75] also reported greater adhesion and peel strength with respect to pure PLA when it was blended with PCL, and Kamrit et al. [9] also observed a decrease in the peel force with increasing PHBV contents in PBS–PHBV blends.

To better understand the changes occurring in the heat-sealed area, FSEM and DSC analyses of the thermo-sealed area (TSA) were carried out. The thermal analysis revealed changes in the crystallisation of polymers as compared with that observed in the initial films. Table 4 shows the melting temperature and enthalpy of PHBV and PCL determined during the first and second heating steps of DSC analyses. In the first heating, which reveals the physical state of polymers in the TSA, the melting temperature and enthalpy of PCL (and so crystallinity) were higher than that observed in the films (64 vs. 53–55 °C and 58–52 vs. 49–50% crystallinity). In contrast, PHBV exhibit more similar T_m_ values in the TSA and the film but lower crystallinity (57–62 vs. 64–68%). This suggests that the annealing produced during heat sealing gave rise to the formation of greater PCL crystals while slightly reducing the PHBV crystallinity by melting the smaller crystals at 120 °C. As expected, in the second heating step, where the previous thermal history was deleted, T_m_ and ΔH_m_ values of PCL were similar in TSA and films. Likewise, for both polymers, the crystallisation temperatures were similar in TSA and films (37 vs. 35–36 C for PCL and 111–113 vs. 110–112 °C for PHBV), while similar supercooling effects were also observed in both cases (ΔT: 20 and 56 °C, respectively, for PCL and PHBV).

**Figure 7:**
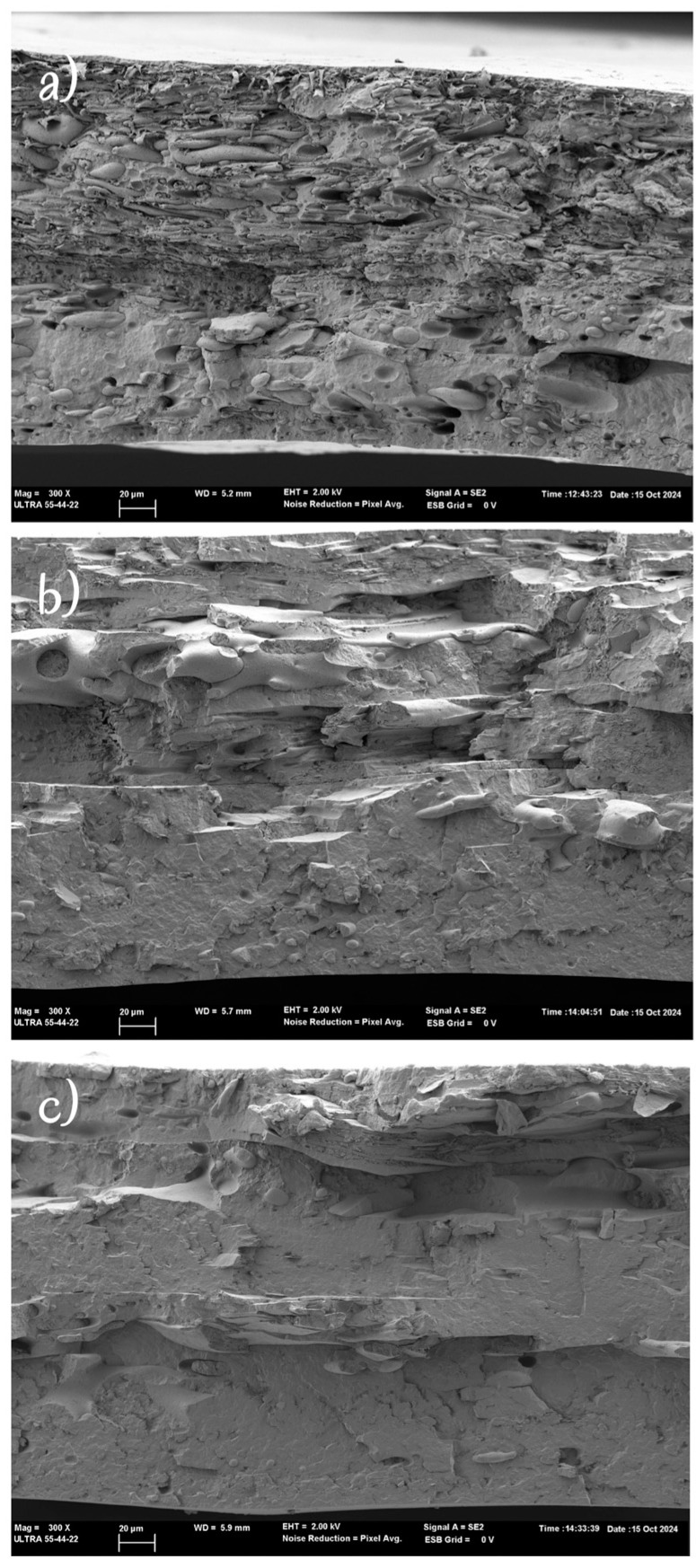
FESEM images of the cross-section of the heat-sealed films: (**a**) 60/40, (**b**) 50/50, and (**c**) 40/60. Magnification: 300×.

Figure 7 shows the FESEM micrographs of the cross-section of the TSA. Both film layers appeared strongly adhered, while the morphology of the layers changed due to the annealing effect. This effect was more remarkable in samples with a higher ratio of PCL, where the increase in its crystallinity affected the cryofracture behaviour. Therefore, the heat sealing of PHBV/PCL blends was possible, giving rise to higher values of seal strength when the PCL was the continuous phase in the film matrix. This result represents a very interesting finding for obtaining heat-sealed PHBV bags for food packaging applications, which has not been reported before.

## 4. Conclusions

The blend films of PHBV with PCL in ratios close to 1:1 (60/40, 50/50, and 40/60) exhibited heat sealability at 120 °C, above the melting point of PCL and below that of PHBV. The seal strength was higher, with less variability, in samples where PCL was the continuous phase (0.22–0.29 N/mm) than in films at a 60/40 ratio (0.14 N/mm), where PHBV was the continuous phase. PCL melting was the responsible for the heat sealing of materials, given that the PCL chains entangle at the interfacial area. Therefore, the continuity of PCL in the matrix guarantees the good thermal adhesion of the materials, making these blend films useful to obtain biodegradable bags for food packaging applications.

These blend films showed lower gas and water vapour barrier capacity than pure PHBV, mainly when the PCL ratio rose and acquired continuity in the films. The 60% PCL resulted in the highest permeability values, which only increased by 140% for oxygen compared with an increase of 600 and 2800% for water vapour and D-limonene, respectively. The blended films also become less stiff (EM reduced by 50% at 60% PCL) and resistant to fracture (TS reduced by 54% at 60% PCL) and were more extensible (E increased by 48% at 60% PCL) than PHBV films. These effects were promoted in the 50/50 and 40/60 films, where PCL was a continuous matrix. Nevertheless, their functional properties still were in the range that is useful for food packaging. The 50/50 blend with a bicontinuous phase of both polymers provided the best balance between heat sealability and barrier properties, making it the most suitable for food packaging in sealed bags. Further studies are required to verify the ability of these materials to preserve foods, depending on their sensitivity to oxidation and moisture or aroma migration. Likewise, the compostability of the materials should be analysed in order to ensure their sustainability.

## Figures and Tables

**Table 1:** Thermal properties (mean values ± standard deviation) obtained from the first and second DSC heating of pure polymers and blends at different PHBV/PCL ratios: melting temperature (T_m_), melting enthalpy (ΔH_m_), and degree of crystallinity (χ_c_).

**1st Heating**						
**Formulation**	**T_m_ PCL**	**∆Hm (J/gPCL)**	**χ_c_ PCL** **(%)**	**T_m_ PHBV**	**∆H_m_ (J/gPHBV)**	**χ_c_ PHBV (%)**
PCL	55 ± 2 ^a^	68 ± 3 ^a^	49 ± 3 ^a^	--	--	--
40–60	53 ± 2 ^a^	68 ± 6 ^a^	49 ± 4 ^a^	168 ± 1 ^b^	88 ± 1 ^a^	67 ± 1 ^a^
50–50	54 ± 1 ^a^	71 ± 1 ^a^	51 ± 1 ^a^	169 ± 1 ^ab^	85 ± 5 ^a^	64 ± 4 ^a^
60–40	55 ± 3 ^b^	69 ± 1 ^a^	50 ± 1 ^a^	171 ± 1 ^ab^	86 ± 4 ^a^	65 ± 3 ^a^
PHBV	--		--	171 ± 0 ^a^	90 ± 0 ^a^	68 ± 0 ^a^
**2nd Heating**						
**Formulation**	**T_m_ PCL**	**∆Hm (J/gPCL)**	**χ_c_ PCL** **(%)**	**T_m_ PHBV**	**∆H_m_ (J/gPHBV)**	**χ_c_ PHBV (%)**
PCL	55.2 ± 0.1 ^b^	68.8 ± 1.4 ^ab^	49.4 ± 1.0 ^ab^	--	--	--
40–60	55.8 ± 0.1 ^ab^	69.1 ± 0.9 ^a^	49.6 ± 0.6 ^a^	166.6 ± 0.2 ^b^	93.7 ± 1.9 ^a^	71.0 ± 1.5 ^a^
50–50	56.0 ± 0.2 ^ab^	62.7 ± 1.9 ^b^	45.0 ± 1.3 ^b^	166.9 ± 0.0 ^b^	90.7 ± 0.2 ^ab^	68.7 ± 0.2 ^ab^
60–40	56.8 ± 1.0 ^a^	66.8 ± 1.7 ^ab^	48.0 ± 1.2 ^ab^	167.7 ± 0.9 ^b^	88.7 ± 1.9 ^b^	67.2 ± 1.4 ^b^
PHBV	--		--	169.7 ± 0.6 ^a^	94.4 ± 0.1 ^a^	71.5 ± 0.1 ^a^

Different letters ^a^ and ^b^ indicate significant differences between samples.

**Table 2:** Water vapour permeability (WVP), D-limonene permeability (LP), and oxygen permeability (OP) and mechanical properties (elastic modulus (EM), str (TS), and elongation at break (E)) of PHBV and PCL films together with PHBV/PCL blends at 60/40, 50/50, and 40/60 ratios.

Film	WVP·10^12^	LP·10^14^	OP·10^13^	EM	TS	E
	(g/m·s·Pa)	(g/Pa·s·m)	(cm^3^/m·s·Pa)	(MPa)	(MPa)	(%)
PHBV	4.1 ± 0.4 ^c^	1.1 ± 0.5 ^d^	2.2 ± 0.2 ^b^	1350 ± 110 ^a^	33 ± 5 ^a^	2.7 ± 0.9 ^b^
60/40	5.9 ± 0.8 ^bc^	6.7 ± 1.8 ^c^	2.4 ± 0.2 ^b^	1030 ± 120 ^b^	17 ± 2 ^b^	1.8 ± 0.3 ^c^
50/50	13.0 ± 5.0 ^b^	18.1 ± 1.2 ^b^	5.1 ± 0.4 ^a^	700 ± 200 ^c^	12 ± 4 ^c^	2.7 ± 0.6 ^b^
40/60	29.0 ± 7.0 ^a^	32.0 ± 9.0 ^a^	5.3 ± 0.4 ^a^	560 ± 60 ^c^	15 ± 2 ^bc^	4.0 ± 0.8 ^a^
PCL	84.5 ± 13.3	185.0 ± 20.0	114.6 ± 13.4	190 ± 30	--	--

The different letters (^a^, ^b^, ^c^…) represent significant differences between the samples containing PHBV.

**Table 3:** Colour coordinates (L*a*b*, Cab*, hab*) of the PHBV and PCL films and of their blends 40:60, 50:50, and 60:40.

	L*	C_ab_*	h_ab_*
PHBV	75.3 ± 3.1 ^c^	19.4 ± 0.2 ^a^	80.0 ± 2.0 ^b^
60–40	75.9 ± 0.7 ^c^	17.8 ± 0.4 ^b^	81.2 ± 0.8 ^b^
50–50	77.6 ± 0.2 ^b^	16.8 ± 0.3 ^c^	81.9 ± 0.2 ^b^
40–60	78.1 ± 0.3 ^b^	15.4 ± 0.3 ^d^	82.5 ± 0.1 ^b^
PCL	89.7 ± 0.8 ^a^	1.6 ± 0.2 ^e^	237.5 ± 51.8 ^a^

The different letters (^a^, ^b^, ^c^…) represent significant differences between the samples.

**Table 4:** Melting temperature (T_m_), enthalpy (ΔH_m_), and degree of crystallinity (χ_c_) of the polymers in the heat-sealed area of the different PHBV/PCL blend films, obtained from the first and second DSC heating steps.

**1st Heating**						
**Formulation**	**T_m_ PCL**	**∆** **Hm (J/gPCL)**	**χ_c_** **PCL** **(%)**	**T_m_ PHBV**	**∆** **H_m_ (J/gPHBV)**	**χ_c_** **PHBV (%)**
40–60	64.0 ± 0.9 ^a^	81.0 ± 3.0 ^a^	58.0 ± 2.0 ^a^	170.0 ± 0.5 ^a^	82.1 ± 2.2 ^a^	62.2 ± 1.7 ^a^
50–50	63.8 ± 0.5 ^a^	79.3 ± 0.4 ^a^	56.9 ± 0.3 ^a^	170.0 ± 1.1 ^a^	80.2 ± 1.9 ^ab^	60.8 ± 1.4 ^ab^
60–40	63.6 ± 0.5 ^a^	73.0 ± 4.0 ^b^	52.0 ± 3.0 ^b^	170.1 ± 0.6 ^a^	79.0 ± 3.0 ^b^	57.0 ± 3.0 ^b^
**2nd Heating**						
**Formulation**	**T_m_ PCL**	**∆** **Hm (J/gPCL)**	**χ_c_** **PCL** **(%)**	**T_m_ PHBV**	**∆** **H_m_ (J/gPHBV)**	**χ_c_** **PHBV (%)**
40–60	56.8 ± 0.1 ^a^	68.0 ± 1.5 ^a^	48.8 ± 1.0 ^a^	167.6 ± 0.2 ^a^	84.0 ± 3.0 ^ab^	63.0 ± 3.0 ^ab^
50–50	56.7 ± 0.5 ^a^	64.1 ± 2.0 ^a^	47.5 ± 1.4 ^a^	167.7 ± 0.4 ^a^	86.0 ± 4.0 ^a^	65.0 ± 3.0 ^a^
60–40	57.0 ± 0.3 ^a^	55.0 ± 4.0 ^b^	47.0 ± 3.0 ^b^	168.1 ± 0.2 ^a^	79.6 ± 1.9 ^b^	60.3 ± 1.4 ^b^

The different letters (^a^, ^b^) represent significant differences between the samples.

## Data Availability

The original contributions presented in this study are included in the article. Further inquiries can be directed to the corresponding authors.
